# Fenofibrate Treatment Inhibits Very-Low-Density Lipoprotein Transport Vesicle Formation by Reducing Sar1b Protein Expression

**DOI:** 10.3390/ijms26104720

**Published:** 2025-05-15

**Authors:** Kayli Winterfeldt, Fahim Rejanur Tasin, Vandana Sekhar, Shadab A. Siddiqi

**Affiliations:** Division of Metabolic & Cardiovascular Sciences, Burnett School of Biomedical Sciences, College of Medicine, University of Central Florida, 6900 Lake Nona Blvd., Room# 349, Orlando, FL 32827, USA; kayli.winterfeldt@ucf.edu (K.W.); fahimrejanur.tasin@ucf.edu (F.R.T.); vandana.sekhar@ucf.edu (V.S.)

**Keywords:** very low-density lipoprotein (VLDL), endoplasmic reticulum (ER), Golgi, fenofibrate, atherosclerosis, intracellular trafficking, coat protein complex II (COPII)

## Abstract

Dyslipidemia is a well-known risk factor in the development and progression of atherosclerosis. VLDL plays a crucial role in maintaining lipid homeostasis; however, even minor fluctuations in its production, intracellular trafficking, and secretion can contribute to the progression of atherosclerosis. Fenofibrate is an FDA-approved drug that effectively lowers plasma triglycerides and VLDL-associated cholesterol while simultaneously increasing HDL levels. Although fenofibrate is a known PPARα agonist with several proposed mechanisms for its lipid-altering effects, its impact on the intracellular trafficking of VLDL has not yet been investigated. We observed that treatment of HepG2 cells with 50 µM of fenofibrate resulted in a significant reduction in VLDL secretion, as evidenced by a significant decrease in the secretion of ^3^H-labeled TAG, fluorescent TAG, and ApoB100 protein into the media. Using confocal microscopy to monitor VLDL intracellular trafficking, we observed a distinct change in VLDL triglyceride localization, suggesting delayed transport through the endoplasmic reticulum and Golgi. An immunoblot analysis revealed a decrease in Sar1B protein expression, a key regulator of COPII protein recruitment, which is essential for VTV formation and intracellular VLDL trafficking, the rate-limiting step in VLDL secretion. Our data reveal a novel mechanism by which fenofibrate alters the lipid profile by interfering with intracellular VLDL trafficking in hepatocytes.

## 1. Introduction

Atherosclerosis is one of the major underlying causes of cardiovascular disease, which is the leading cause of death worldwide. It is well established that lipids play a vital role in contributing to the development and progression of atherosclerosis through abnormal secretion and the modification of very-low-density lipoprotein (VLDL), which contributes to the formation of the fatty streak [1,2,3,4]. Fenofibrate is an FDA-approved drug used to treat dyslipidemia. It is a known agonist of peroxisome proliferator-activated receptor alpha (PPARα), which functions as a transcription factor to regulate the expression of various genes associated with lipid metabolism [5,6,7]. Fenofibrate has been widely utilized and has been shown to significantly reduce levels of plasma triglycerides and VLDL, while high-density lipoprotein (HDL) levels are increased, improving the overall cholesterol profile [8,9,10,11,12].

Several mechanisms of action have been proposed for the lipid-altering effects of fenofibrate. It has been shown that in response to fenofibrate, there is a significant shift in the body’s ability to process VLDL through increases in lipoprotein lipase activity seen through increased activation of ApoAV and decreased expression of ApoCIII, which is a known LPL inhibitor [5,13,14,15]. It has also been shown to have a significant effect on the body’s ability to uptake fatty acids derived from VLDL into peripheral tissues through the regulation of VLDL receptors, as well as by reducing the concentration of small, dense LDL particles in favor of larger, less dense LDL particles, which have a higher binding affinity for receptors [13,15,16]. Several studies have also shown that PPARα agonists increase the expression of genes associated with fatty acid oxidation [17,18,19,20], as well as inhibiting de novo fatty acid synthesis [5,6,8,15]. An increase in fatty acid oxidation in combination with a decrease in de novo fatty acid synthesis in hepatocytes would decrease the availability of free fatty acids for triglyceride synthesis and consequently the synthesis and secretion of VLDL.

The biogenesis of VLDL begins with the co-translational translocation of ApoB100 into the endoplasmic reticulum (ER). Upon its entry into the ER, ApoB100 interacts with microsomal triglyceride transferase protein (MTP), which facilitates its partial lipidation, leading to the formation of primordial VLDL particles [21,22]. Once formed in the ER lumen, primordial VLDL exits the ER in a specialized transport vesicle called the VLDL transport vesicle (VTV). The biogenesis of VTVs from the ER requires the successful recruitment of COPII proteins [23,24,25]. Conventional COPII-dependent vesicle formation is initiated by the conversion of Sar1B-GDP to Sar1B-GTP, a process catalyzed by the guanine nucleotide exchange factor (GEF) Sec12, which enables Sar1B-GTP to bind with the ER membrane. This binding event triggers the sequential recruitment of the Sec23/24 heterodimer, followed by the Sec13/31 complex, resulting in membrane deformation and the eventual budding and release of the vesicle [26,27,28,29]. While VTV formation has been shown to be COPII-dependent, additional studies have demonstrated that proteins unique to the VTV—such as SVIP [30], RTN3 [31], and CideB [32]—are also required for its biogenesis [30,31,32]. The VTV travels from the ER and fuses with the Golgi apparatus for the further maturation of VLDL particles. While the proteins and signaling pathways involved in this stage are not yet fully understood, it is known that VLDL undergoes glycosylation and phosphorylation within the Golgi [22,33,34]. The question of whether VLDL undergoes further lipidation at this stage remains a topic of ongoing debate [23,35,36,37,38]. Once mature VLDL is formed, it exits the Golgi in a specialized transport vesicle known as the post-Golgi VLDL transport vesicle (PG-VTV), which then travels to and fuses with the plasma membrane for the eventual secretion of VLDL [39,40].

VLDL synthesis and trafficking is a tightly regulated process, and while numerous studies have examined the effects of fenofibrates on VLDL secretion, it has not been previously investigated whether fenofibrate directly affects the intracellular trafficking of VLDL particles in hepatocytes. In this study, we aimed to determine whether fenofibrate affects VLDL secretion by interfering with the intracellular trafficking pathway. Our findings reveal a significant alteration in VLDL trafficking and suggest an additional mechanism underlying the lipid-lowering effects of fenofibrate.

## 2. Results

### 2.1. Validating Fenofibrates Effect on VLDL Secretion

**Figure 1:**
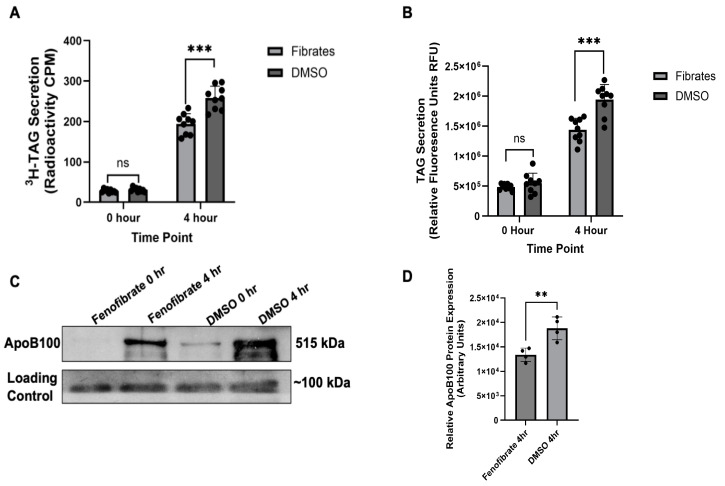
The VLDL secretion analysis. (**A**) Radioactivity ^3^H-TAG secretion assay, following treatment of HepG2 cells with fenofibrates vs. DMSO at 0 and 4 h post-treatment. The data analysis was performed through GraphPad PRISM, with a *t*-test showing no significance at 0 h with a ns *p* value of 0.2071 and significance at 4 h with a *** *p* value of 0.0001, *n* ≥ 9. (**B**) Fluorescent TopFluor secretion assay, following treatment of HepG2 cells with fenofibrate vs. DMSO at 0 and 4 h post-treatment. The data analysis was performed through GraphPad PRISM, with a *t*-test showing no significance at 0 h with a ns *p* value of 0.3062 and significance at 4 h with a *** *p* value of 0.0002, *n* ≥ 9. (**C**) Immunoblot of secreted ApoB100 and a non-specific band at ~100 kDa used as a loading control; *n* ≥ 4. (**D**) Changes in ApoB100 protein expression were quantified through an NIH ImageJ analysis and a data analysis was performed through GraphPad PRISM, with a *t*-test showing significance at 4 h post-treatment with a ** *p* value of 0.0070; *n* ≥ 4.

VLDL secretion in response to fenofibrate treatment was monitored using three distinct methods: ^3^H-tagged TAG secretion, TopFluor-tagged TAG secretion, and ApoB100 secretion. For TAG secretion, it is expected that as the tagged fatty acid is taken up by the cell and incorporated into VLDL, the secreted particles will retain the respective tag, which can then be measured to quantify VLDL secretion. For both assays, HepG2 cells were treated through a pulse–chase approach, with a one hour pulse treatment of oleic acid (OA) in combination with either 50 µM of fenofibrate or DMSO. Following the pulse, the treatment medium was removed and replaced with OA-free medium containing the same respective treatment—either 50 µM of fenofibrate or DMSO. Both the radioactivity-based and fluorescent assays showed a significant decrease in VLDL secretion at 4 h post-pulse, which is consistent with the known maximum absorption of fenofibrate occurring between 4 and 6 h [12,41]. In the time course experiments conducted during the optimization stage, we observed the most significant reduction in VLDL secretion at the 4 h timepoint, which led to its selection for subsequent experiments. Using both the ^3^H-TAG and TopFluor TAG assays, we observed 20–30% decreases in VLDL secretion when the cells were treated with 50 µM of fenofibrate compared to DMSO (Figure 1A,B).

The levels of ApoB100 in the cell culture media were also monitored to validate the VLDL secretion. As the primary structural protein of VLDL, changes in VLDL secretion are expected to correlate with alterations in the concentration of secreted ApoB100. Following the observed change in TAG secretion at 4 h post-treatment, as shown in Figure 1A,B, we conducted an immunoblot analysis on the collected cell culture media to further confirm that fenofibrate affects VLDL secretion. A significant decrease in ApoB100 protein levels was observed in the fibrate treatment group compared to the DMSO group, with an average reduction of 31%, as determined through protein quantification using an NIH ImageJ analysis, version 10 (Figure 1C,D).

**Figure 2:**
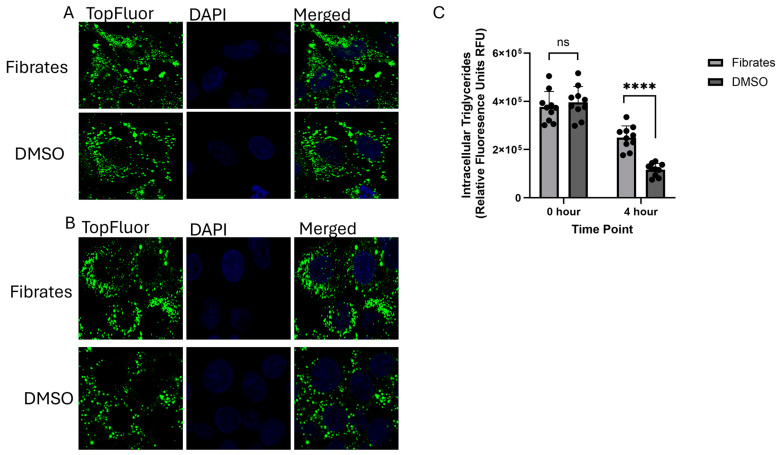
Intracellular VLDL TopFluor intensity: (**A**) confocal microscopy of fenofibrate-treated vs. DMSO-treated HepG2 cells at 0 h post-treatment at 100× magnification; (**B**) confocal microscopy of fenofibrate-treated vs. DMSO-treated HepG2 cells 4 h post-treatment at 100× magnification; (**C**) a statistical analysis of the intracellular fluorescence intensity of the TopFluor signal was quantified through NIH ImageJ and a data analysis was performed through GraphPad PRISM. No significance was identified at the 0 h timepoint with a ns *p* value of 0.5054. However, significance was identified at the 4 h timepoint with a **** *p* value of <0.0001; *n* ≥ 10.

In addition to monitoring VLDL secretion, the fluorescently tagged TopFluor OA enabled us to monitor the intracellular trafficking of VLDL particles using confocal microscopy. At 0 h post-treatment, the fibrate-treated cells and the DMSO-treated cells showed no significant difference in the levels of intracellular fatty acids associated with the TopFluor signal. This indicates that treatment with fenofibrates does not affect the cells’ ability to uptake free fatty acids (Figure 2A,C). However, at 4 h post-treatment, there was a significantly higher concentration of intracellular fluorescence associated with TopFluor in the fibrate-treated cells compared to the DMSO-treated cells (Figure 2B,C). This increase in intracellular intensity in combination with the decrease in secreted VLDL shows that fenofibrate significantly reduces VLDL secretion in HepG2 cells.

### 2.2. Monitoring the Effects of Fenofibrate on Intracellular VLDL Trafficking

**Figure 3:**
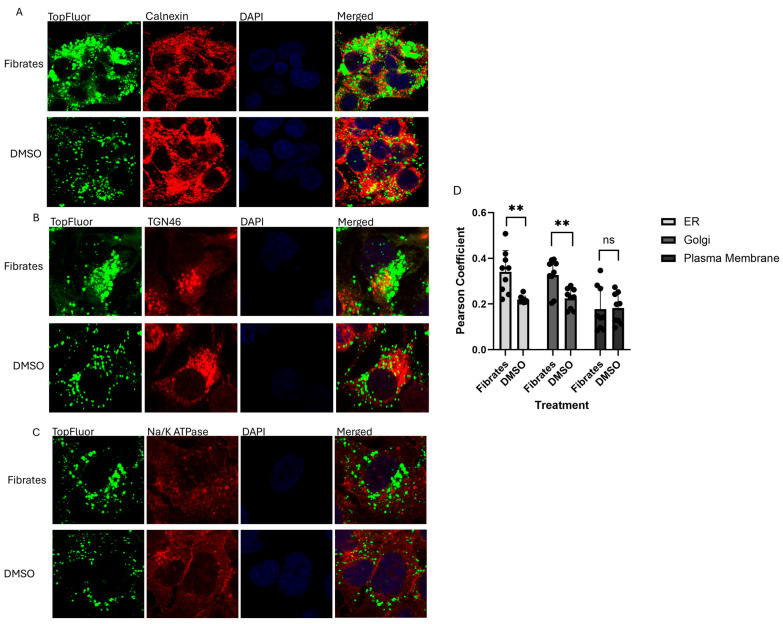
A colocalization analysis of confocal microscopy of fenofibrate-treated vs. DMSO-treated HepG2 cells at 4 h post-treatment: (**A**) TopFluor colocalization with the ER using calnexin staining at 100× magnification; (**B**) TopFluor colocalization with the Golgi using TGN46 staining at 100× magnification; (**C**) TopFluor colocalization with the plasma membrane through Na/K ATPase staining at 100× magnification; (**D**) a statistical analysis of colocalization, whereby the Pearson coefficient was calculated through NIH ImageJ and statistical significance was determined through GraphPad PRISM. Significance was determined in the ER and Golgi with ** *p* values of 0.0013 and 0.0024, respectively. No significance was found in the plasma membrane with a ns *p* value of 0.9087, *n* ≥ 8.

In our recently published study, we demonstrated that TopFluor OA colocalizes with the ER, Golgi, and plasma membrane during VLDL trafficking and secretion, thereby establishing it as a valuable tool for monitoring the various stages of intracellular VLDL transport [42]. HepG2 cells were treated with 50 µM of fenofibrate or DMSO as a control and incubated with TopFluor OA. The immunostaining and confocal microscopy at 4 h post-treatment revealed a significant increase in colocalization of TopFluor with the ER, as visualized through calnexin staining (Figure 3A,D), as well as with the Golgi, as indicated by TGN46 staining, compared to the DMSO-treated cells at the same timepoint (Figure 3B,D). There was no significant difference in colocalization in the plasma membrane (Figure 3C,D). These data suggest that fibrate treatment directly impacts the intracellular trafficking of VLDL, causing a delay in VLDL secretion that begins in the ER and persists through the later stages of trafficking in the Golgi.

### 2.3. Determining the Cause of the Intracellular VLDL Trafficking Defect

**Figure 4:**
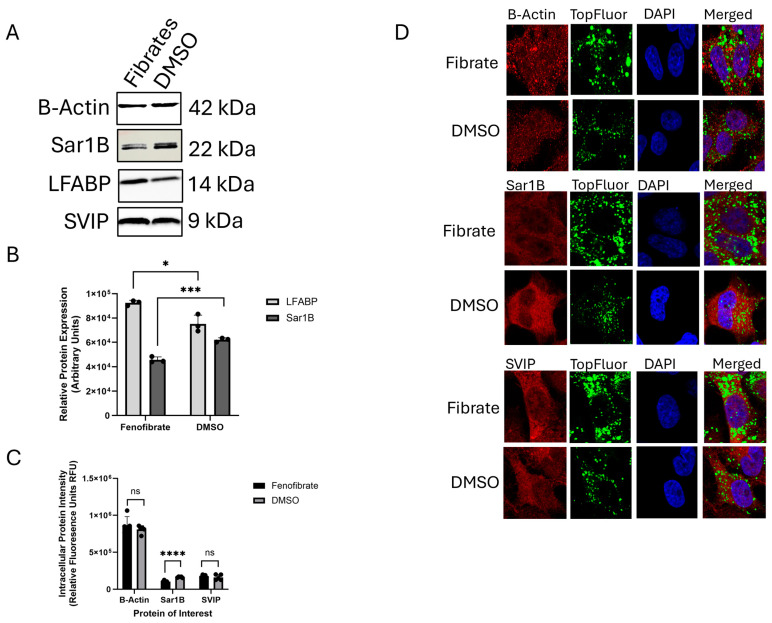
Determining the cause of the intracellular trafficking defect. (**A**) Immunoblots of β-Actin, Sar1B, LFABP, and SVIP in fenofibrate-treated cells vs. DMSO-treated cells. (**B**) Changes in LFABP and Sar1B protein expression were quantified using NIH ImageJ and a data analysis was performed using GraphPad PRISM. The changes in LFABP and Sar1B were shown to be significant with *, *** *p* values of 0.0162 and 0.0006, respectively; *n* ≥ 3. (**C**) The fluorescence intensity of proteins of interest was quantified through NIH ImageJ and a statistical analysis was performed through GraphPad PRISM. No significant change in protein expression was found for β-Actin or SVIP with ns *p* values of 0.3425 and 0.3422, respectively. Significance was found for Sar1B with a **** *p* value of <0.0001; *n* ≥ 5. (**D**) Confocal images of Sar1B, β-Actin, and SVIP following fenofibrate vs. DMSO treatment at 100× magnification.

To investigate the cause of the VLDL trafficking defect, HepG2 cells were co-treated with 0.4 mM of BSA-OA and either 50 µM of fenofibrate or DMSO for 1 h. Following this initial treatment, the OA-containing medium was removed, and the cells were incubated for an additional 4 h in media containing either fenofibrate or DMSO. The cell lysates were collected and analyzed using immunoblotting to assess the expression of key proteins involved in VLDL trafficking, including Sar1B, LFABP, and SVIP. A significant reduction in Sar1B protein levels—averaging 29%—was observed in the fenofibrate-treated group compared to the DMSO control. In contrast, no significant changes were detected in SVIP expression or in β-Actin, which served as the loading control (Figure 4A,B). This trend was further supported by immunofluorescence staining and confocal microscopy (Figure 4B,C). As additional validation of the treatment efficacy, we observed a significant increase in LFABP expression—consistent with previous findings that fenofibrate, a known PPARα agonist, upregulates LFABP. This increase was confirmed both at the protein level (Figure 4A,B) and at the transcript level using qPCR (Supplemental Figure S1).

## 3. Discussion

VLDL plays a critical role in maintaining lipid homeostasis, and even minor alterations in its synthesis and secretion rates can have significant health implications, including the onset and progression of atherosclerosis [4,43,44,45]. Atherosclerosis, in turn, is the primary underlying cause of cardiovascular disease—the leading cause of death globally. Given that atherosclerosis remains a major global health concern, the treatment strategies often emphasize lifestyle modifications such as weight loss [2,4,46,47]. The pharmacological interventions typically target VLDL production and secretion to reduce circulating pro-atherogenic particles; however, this approach may inadvertently cause increased hepatic lipid accumulation, raising the risk of non-alcoholic fatty liver disease (NAFLD) [2]. Fenofibrate, a PPARα agonist, is an FDA-approved drug used in the treatment of hypertriglyceridemia, which is a major risk factor in the development of atherosclerosis. Several mechanisms of action have been proposed for the lipid lowering effects of fenofibrate, including increased rates of β-oxidation, decreased rates of de novo lipogenesis, and changes in the way that the body is able to process VLDL [13,14,15,16,17,18,19,20]; however, no studies have been performed to determine whether, in addition to these methods, fenofibrate directly affects the intracellular trafficking of VLDL.

This study confirmed that in response to fenofibrate treatment, there was a significant decrease in VLDL secretion 4 h post-treatment, which aligns with the known max absorption of the drug. This reduction was validated through TAG-based radioactivity and fluorescence assays, as well as via ApoB100 secretion assessed by immunoblotting. Additionally, using the fluorescently tagged TopFluor OA and confocal microscopy, we monitored the intracellular trafficking of VLDL. A significant increase in colocalization was observed between the ER and Golgi in the fenofibrate-treated cells compared to the DMSO-treated controls, indicating that the drug interferes with normal VLDL trafficking and maturation at these stages. To explore the underlying cause of this trafficking defect, we performed immunoblotting to assess changes in key regulatory proteins, revealing a significant decrease in Sar1B expression, which was further corroborated via confocal microscopy.

Sar1B is the initiating protein of COPII coat recruitment, which as previously mentioned is a known requirement for VLDL trafficking and is a part of the rate-limiting step in VLDL intracellular trafficking and secretion [22,30,31,32,33,34,35,36,37]. Sar1B recruits sec12, a guanine exchange factor that activates Sar1B, causing binding to the ER membrane. Sar1B binding then recruits heterodimers of sec23/24 followed by sec 13/31, leading to membrane deformation and eventual vesicle release [22]. A decrease in Sar1B protein expression would explain the delay in VLDL trafficking from the ER, as it would lead to a decrease in COPII protein recruitment required for the formation of the VTV, meaning primordial VLDL particles would be trapped in the ER. The later steps of VLDL trafficking are not as well defined. Many of the signaling pathways and regulatory proteins involved in the later aspects have yet to be elucidated. A recent study by our lab identified LFABP as a key regulatory protein in the Golgi stage of VLDL trafficking [42]. It was found that in response to LFABP knockdown, there was a significant decrease in VLDL secretion [42]. Upon further investigation using TopFluor OA, it was found that there was increased accumulation of VLDL within the Golgi compared to the control cells [42]. The exact role of LFABP in VLDL trafficking remains unknown; however, previous studies have hypothesized that LFABP could play a role in generating the larger vesicles required for lipoprotein transport. It has been previously shown that in chylomicron transport in the intestines, LFABP displayed budding activity off the ER membrane. The study hypothesized that it could play a role in cargo selection or generating larger vesicles [48]. For comparison, typical COPII protein transport vesicles range in size from 55 to 70 nm in diameter, while pre-chylomicron transport vesicles are known to be 250 nm in diameter. Comparing that to VLDL transport vesicles, the VTV is approximately 110 nm, while the PG-VTV range is 300–350 nm. It is possible that LFABP plays a similar role in VLDL trafficking and could aid in forming the larger PG-VTV. However, treatment through fenofibrate leads to an increase in LFABP expression. Previous experiments have shown that in response to fenofibrate treatment, there is an increase in β-oxidation; it is possible that in response to fenofibrate treatment there are other signaling pathways that are activated for LFABP to prioritize transporting FAs for β-oxidation instead of VLDL secretion, thereby leading to this buildup in the Golgi. More research is needed to determine the exact cause of this phenomenon or if other unknown proteins are responsible for the Golgi defect.

VLDL trafficking and secretion is a tightly regulated processes crucial for maintaining lipid homeostasis. However, even small alterations in the rates of VLDL transport and secretion can lead to dyslipidemia, contributing to the development of atherosclerosis and other metabolic conditions. While numerous studies have explored the lipid-lowering effects of fenofibrates, this study is the first to propose an additional mechanism of action that directly impacts intracellular VLDL trafficking, subsequently reducing VLDL secretion from the liver.

## 4. Materials and Methods

### 4.1. Cell Culture

The human hepatoma cells (HepG2) were purchased from American type culture collection (HB-8065, ATCC, Manassas, VA, USA). The cells were cultured at 37 °C with 5% CO_2_ in Dulbecco’s modified Eagle’s medium (DMEM, Corning, Corning, NY, USA), which was supplemented with 10% fetal bovine serum (FBS) and 1% penicillin/streptomycin (Gibco, Grand Island, NY, USA). For passaging, the cells were rinsed with sterile phosphate-buffered saline (PBS, Gibco) and incubated with 0.25% Trypsin–EDTA (Gibco) until the cells were fully lifted. Fresh cell culture medium was supplemented back in, and the cells were replated.

### 4.2. Fenofibrate Preparation and Treatment

The fenofibrate was purchased from Thermo Fisher Scientific, Inc. (Waltham, MA, USA). A 50 mM stock solution was prepared by dissolving fenofibrate in DMSO via repeated pipetting. The stock solution was further diluted to the desired concentration in DMEM cell culture medium supplemented with 2.5% or 5% FBS. The control medium was made by substituting fenofibrate for DMSO. The cells were first incubated for 1 h in medium containing 0.1 mM of OA and either 50 µM of fenofibrate or DMSO. Following the incubation, the cells were washed to remove the OA, and fresh medium containing either 50 µM of fenofibrate or DMSO was added. The cells were then incubated for an additional 4 h to measure the secretion.

### 4.3. ^3^H-TAG Secretion Assay

HepG2 cells were seeded in a 6-well plate and grown in DMEM containing 10% FBS until the cells reached 60–70% confluency. The fenofibrate treatment medium was made fresh and supplemented with 0.4 mM of OA containing BSA-OA (Sigma, St. Louis, MO, USA) and ^3^H-tagged OA (Perkin Elmer, Waltham, MA, USA). The 10% FBS DMEM was removed, and the cells were washed with PBS before the addition of 2.5% FBS treatment medium. Following a one hour incubation period, the radioactive medium was properly disposed of, the cells were washed with warm 5% FBS medium, and fresh 5% treatment medium was added back into the cells, which were incubated for various desired timeframes. At the desired timepoints, 250 µL of medium was collected from each well and replaced with identical volumes of fresh treatment media. From the collected media, 100 µL was vortexed with 5 mL of scintillation fluid (SX18-4, Fisher Scientific, Pittsburgh, PA, USA) and the ^3^H TAG CPM result was recorded (Perkin Elmer Tri-Carb 2910 TR)

### 4.4. TopFluor-Tagged Oleic Acid Assay

HepG2 cells were plated and grown on Poly-D-lysine-coated coverslips (Neuvitro, Camas, WA, USA) and allowed to incubate overnight at 37 °C. Here, 2.5% and 5% fenofibrate treatment media were prepared fresh and an OA stock solution containing TopFluor-tagged OA and BSA-OA was added to the 2.5% FBS treatment media to a final concentration of 0.1 mM OA. Prior to treatment, the old cell culture media were removed, and the cells are washed with PBS. Then, 2.5% treatment medium was added to the cells and allowed to incubate for 1 h at 37 °C. The treatment medium was removed, the cells were washed, and 5% FBS treatment medium was supplemented back into the cells, which were incubated for various desired timeframes. At those timepoints, the cell culture medium was collected and measured for FITC fluorescence using an Envision 2104 multilabel reader. The fluorescence data were recorded for a data analysis and the cell culture medium was recollected and utilized as a protein sample for ApoB100 immunoblotting. The coverslips at these timepoints were also fixed and permeabilized for cell staining.

### 4.5. Immunostaining and Confocal Microscopy

The coverslips were washed with PBS and incubated with 4% paraformaldehyde (PFA, Thermo Scientific, Waltham, MA, USA) for 10 min. The PFA was removed and the coverslips were washed with PBS prior to permeabilization with 0.1% Triton X-100 (Sigma, St. Louis, MO, USA) for 10 min. The triton solution was removed and the coverslips were washed with PBS and blocked with 10% goat serum for 30 min. The primary antibodies for colocalization studies for targeting the ER (calnexin, MA3-027), Golgi (TGN46, ab50595) and the plasma membrane (Na/K ATPase, ab283318) were purchased from Invitrogen (Waltham, MA, USA) and Abcam (Cambridge, UK). The primary antibodies for β-Actin (C-4, Santa Cruz Biotechnology, Dallas, TX, USA), Sar1B (Ab155278, Abcam), and SVIP (HPA039807, Sigma) were diluted in 1% bovine serum albumin (BSA, Sigma) in phosphate-buffered saline–Tween20 (PBS-T). The coverslips were incubated with primary antibodies overnight at 4 °C. The coverslips were washed with PBS prior to fluorescent secondary antibody incubation (sc-2780, Santa Cruz Biotechnology; A11005, Invitrogen) for 1 h at room temperature. The coverslips were then washed a final time in PBS before being mounted on microscope slides with DAPI-Fluoromount-G™ clear mounting medium (Southern Biotech, Birmingham, AL, USA). The microscope slides were allowed to dry for 24 h before imaging. Confocal images were taken and processed with Leica TCS SP5II and LAS X office. The fluorescent intensity and colocalization were calculated through NIH ImageJ.

### 4.6. Immunoblotting

#### 4.6.1. ApoB100

Equal volumes of collected cell culture media were added to Laemmli’s buffer (Bio-Rad, Hercules, CA, USA), mixed, boiled for 5 min, centrifuged, loaded into an 8% acrylamide gel, and resolved through SDS-PAGE. The proteins were transferred onto nitrocellulose membranes (Bio-Rad) overnight at 50 mAmps. The following day, the membranes were blocked in 10% milk, washed with PBS-T, and incubated with primary antibodies (ApoB100, sc-13538, Santa Cruz Biotechnology) overnight at 4 °C. The membranes were washed with PBS-T and incubated for 1 h at room temperature with HRP-conjugated secondary antibodies. Protein expression was detected using ECL Reagents (Thermo Scientific, Waltham, MA, USA) and Azure600 (Azure Biosystems, Dublin, CA, USA).

#### 4.6.2. Sar1B, β-Actin, LFABP, and SVIP

The cell lysates were prepared following 1 h of treatment with 2.5% FBS treatment media supplemented with 0.4 mM OA. After a 4 h incubation with 5% FBS treatment media, the cells were washed, scraped, and collected into microcentrifuge tubes, and then the cells were pelleted. The cell culture medium was removed, then RIPA buffer (Thermo Scientific, Waltham, MA, USA) was added directly to the cell pellet and resuspended. The cells were incubated on ice for 5 min and then sonicated and centrifuged for 15 min at 13,000× *g*. The protein concentration of the cell lysate was determined through a Bradford assay using protein assay dye (Bio-Rad) and the absorbance was calculated with a Beckman Coulter DU800 Spectrophotometer. The protein concentration remained constant between the prepared cell lysates when preparing the protein samples for immunoblotting. The protein samples were prepared from the collected cell lysates and Laemmli buffer, then added to 15% acrylamide gels and resolved through SDS-PAGE. The proteins were transferred to nitrocellulose membranes at room temperature for 1 h at 80 mAmps. The membranes were blocked with 10% milk, washed with PBS-T, and incubated with primary antibodies for β-Actin (C-4, Santa Cruz Biotechnology), Sar1B (Ab155278, Abcam), LFABP (C-4, Santa Cruz Biotechnology), and SVIP (HPA039807, Sigma) overnight at 4 °C. The primary antibody was removed, then the membranes were washed and incubated for 1 h at room temperature with HRP-conjugated secondary antibodies. The protein expression was detected using ECL Reagents (Pierce) and Azure600.

### 4.7. Statistical Analysis

A statistical analysis of the data was performed using an unpaired *t*-test through GraphPad PRISM version 10 software.

## Data Availability

All data are presented in the manuscript and raw data (such as for immunoblots) will be available upon request.

## Supplementary Materials

The following supporting information can be downloaded at https://www.mdpi.com/article/10.3390/ijms26104720/s1.

## References

[1] Rafieian-Kopaei M., Setorki M., Doudi M., Baradaran A., Nasri H. (2014). Atherosclerosis: Process, indicators, risk factors and new hopes. Int. J. Prev. Med..

[2] Pahwa R., Jialal I. (2024). Atherosclerosis. StatPearls.

[3] Linton M.F., Yancey P.G., Davies S.S., Jerome W.G., Linton E.F., Song W.L., Doran A.C., Vickers K.C., Feingold K.R., Ahmed S.F., Anawalt B., Blackman M.R., Boyce A., Chrousos G., Corpas E., de Herder W.W., Dhatariya K., Dungan K. (2000). The Role of Lipids and Lipoproteins in Atherosclerosis. Endotext.

[4] Ginsberg H.N., Packard C.J., Chapman M.J., Boren J., Aguilar-Salinas C.A., Averna M., Ference B.A., Gaudet D., Hegele R.A., Kersten S. (2021). Triglyceride-rich lipoproteins and their remnants: Metabolic insights, role in atherosclerotic cardiovascular disease, and emerging therapeutic strategies-a consensus statement from the European Atherosclerosis Society. Eur. Heart J..

[5] Schoonjans K., Staels B., Auwerx J. (1996). The peroxisome proliferator activated receptors (PPARS) and their effects on lipid metabolism and adipocyte differentiation. Biochim. Biophys. Acta.

[6] Schoonjans K., Staels B., Auwerx J. (1996). Role of the peroxisome proliferator-activated receptor (PPAR) in mediating the effects of fibrates and fatty acids on gene expression. J. Lipid Res..

[7] Kersten S. (2008). Peroxisome proliferator activated receptors and lipoprotein metabolism. PPAR Res..

[8] Keating G.M., Croom K.F. (2007). Fenofibrate: A review of its use in primary dyslipidaemia, the metabolic syndrome and type 2 diabetes mellitus. Drugs.

[9] Hiukka A., Leinonen E., Jauhiainen M., Sundvall J., Ehnholm C., Keech A.C., Taskinen M.R. (2007). Long-term effects of fenofibrate on VLDL and HDL subspecies in participants with type 2 diabetes mellitus. Diabetologia.

[10] Chen Y.Q., Zhao S.P., Ye H.J. (2020). Efficacy and safety of coenzyme A versus fenofibrate in patients with hyperlipidemia: A multicenter, double-blind, double-mimic, randomized clinical trial. Curr. Med. Res. Opin..

[11] Chapman M.J. (2003). Fibrates in 2003: Therapeutic action in atherogenic dyslipidaemia and future perspectives. Atherosclerosis.

[12] Balfour J.A., McTavish D., Heel R.C. (1990). Fenofibrate. A review of its pharmacodynamic and pharmacokinetic properties and therapeutic use in dyslipidaemia. Drugs.

[13] Bijland S., Pieterman E.J., Maas A.C., van der Hoorn J.W., van Erk M.J., van Klinken J.B., Havekes L.M., van Dijk K.W., Princen H.M., Rensen P.C. (2010). Fenofibrate increases very low density lipoprotein triglyceride production despite reducing plasma triglyceride levels in APOE*3-Leiden.CETP mice. J. Biol. Chem..

[14] Staels B., Vu-Dac N., Kosykh V.A., Saladin R., Fruchart J.C., Dallongeville J., Auwerx J. (1995). Fibrates downregulate apolipoprotein C-III expression independent of induction of peroxisomal acyl coenzyme A oxidase. A potential mechanism for the hypolipidemic action of fibrates. J. Clin. Investig..

[15] McKeage K., Keating G.M. (2011). Fenofibrate: A review of its use in dyslipidaemia. Drugs.

[16] Gao Y., Shen W., Lu B., Zhang Q., Hu Y., Chen Y. (2014). Upregulation of hepatic VLDLR via PPARalpha is required for the triglyceride-lowering effect of fenofibrate. J. Lipid Res..

[17] Minnich A., Tian N., Byan L., Bilder G. (2001). A potent PPARalpha agonist stimulates mitochondrial fatty acid beta-oxidation in liver and skeletal muscle. Am. J. Physiol. Endocrinol. Metab..

[18] Ip E., Farrell G.C., Robertson G., Hall P., Kirsch R., Leclercq I. (2003). Central role of PPARalpha-dependent hepatic lipid turnover in dietary steatohepatitis in mice. Hepatology.

[19] Harano Y., Yasui K., Toyama T., Nakajima T., Mitsuyoshi H., Mimani M., Hirasawa T., Itoh Y., Okanoue T. (2006). Fenofibrate, a peroxisome proliferator-activated receptor alpha agonist, reduces hepatic steatosis and lipid peroxidation in fatty liver Shionogi mice with hereditary fatty liver. Liver Int..

[20] Staels B., Dallongeville J., Auwerx J., Schoonjans K., Leitersdorf E., Fruchart J.C. (1998). Mechanism of action of fibrates on lipid and lipoprotein metabolism. Circulation.

[21] Feingold K.R., Feingold K.R., Ahmed S.F., Anawalt B., Blackman M.R., Boyce A., Chrousos G., Corpas E., de Herder W.W., Dhatariya K., Dungan K. (2000). Introduction to Lipids and Lipoproteins. Endotext.

[22] Tiwari S., Siddiqi S.A. (2012). Intracellular trafficking and secretion of VLDL. Arterioscler. Thromb. Vasc. Biol..

[23] Tran K., Thorne-Tjomsland G., DeLong C.J., Cui Z., Shan J., Burton L., Jamieson J.C., Yao Z. (2002). Intracellular assembly of very low-density lipoproteins containing apolipoprotein B100 in rat hepatoma McA-RH7777 cells. J. Biol. Chem..

[24] Siddiqi S.A. (2008). VLDL exits from the endoplasmic reticulum in a specialized vesicle the VLDL transport Vesicle, in rat primary hepatocytes. Biochem. J..

[25] Kuge O., Dascher C., Orci L., Rowe T., Amherdt M., Plutner H., Ravazzola M., Tanigawa G., Rothman J.E., Balch W.E. (1984). Sar1B promotes vesicle budding from the endoplasmic reticulum but not Golgi compartments. J. Cell Biol..

[26] Aridor M., Fish K.N., Bannykn S., Weissman J., Roberts T.H., Lippincott-Schwartz J., Balch W.E. (2001). The Saar1GTPase Coordinates biosynthetic cargo selection with endoplasmic reticulum export site assembly. J. Cell Biol..

[27] Barlowe C., Schekman R. (1993). SEC12 encodes a guanine-nucleotide-exchange factor essential for transport vesicle budding from the ER. Nature.

[28] Jenson D., Schekman R. (2011). COPII- mediated vesicle formation at a glance. J. Cell Sci..

[29] Kuehn M.J., Herrmann J.M., Schekman R. (1998). COPII_ cargo interactions direct protein sorting into ER- derived transport vesicles. Nature.

[30] Tiwari S., Siddiqi S., Zhelyabovska O., Siddiqi S.A. (2016). Silencing of Small Valosin-containing Protein-interacting Protein (SVIP) Reduces Very Low-Density Lipoprotein (VLDL) Secretion from Rat Hepatocytes by Disrupting Its Endoplasmic Reticulum (ER)-to-Golgi Trafficking. J. Biol. Chem..

[31] Tiwari S., Siddiqi S., Siddiqi S.A. (2013). CideB protein is required for the biogenesis of very low-density lipoprotein (VLDL) transport vesicle. J. Biol. Chem..

[32] Siddiqi S., Zhelyabovska O., Siddiqi S.A. (2018). Reticulon 3 regulates very low-density lipoprotein secretion by controlling very low-density lipoprotein transport vesicle biogenesis. Can. J. Physiol. Pharmacol..

[33] Swift L.L. (1996). Role of the Golgi apparatus in the phosphorylation of apolipoprotein B. J. Biol. Chem..

[34] Vukmirica J., Nishimaki-Mogami T., Tran K., Shan J., McLeod R.S., Yuan J., Yao Z. (2002). The N-linked oligosaccharides at the amino terminus of human apoB are important for the assembly and secretion of VLDL. J. Lipid Res..

[35] Alexander C.A., Hamilton R.L., Havel R.J. (1976). Subcellular localization of B apoprotein of plasma lipoproteins in rat liver. J. Cell Biol..

[36] Bamberger M.J., Lane M.D. (1990). Possible role of the Golgi apparatus in the assembly of very low-density lipoprotein. Proc. Natl. Acad. Sci. USA.

[37] Gusarova V., Brodsky J.L., Fisher E.A. (2003). Apolipoprotein B100 exit from the endoplasmic reticulum (ER) is COPII-dependent, and its lipidation to very low-density lipoprotein occurs post-ER. J. Biol. Chem..

[38] Rusinol A., Verkade H., Vance J.E. (1993). Assembly of rat hepatic very low-density lipoproteins in the endoplasmic reticulum. J. Biol. Chem..

[39] Hossain T., Riad A., Siddiqi S., Parthasarathy S., Siddiqi S.A. (2014). Mature VLDL triggers the biogenesis of a distinct vesicle from the trans-Golgi network for its export to the plasma membrane. Biochem. J..

[40] Siddiqi S.A. (2015). In Vitro Analysis of the Very-Low Density Lipoprotein Export from the Trans-Golgi Network. Curr. Protoc. Cell Biol..

[41] Sidhu G., Tripp J. (2025). Fenofibrate. StatPearls.

[42] Winterfeldt K., Rejanur Tasin F., Siddiqi S.A. (2025). Establishing the Role of Liver Fatty Acid-Binding Protein in Post-Golgi Very-Low-Density Lipoprotein Using a Novel Fluoresence Based Assay. Int. J. Mol. Sci..

[43] Arving A., Osganian S.A., Cohen D.E., Corey K.E., Feingold K.R., Anawalt B., Blackman M.R., Boyce A., Chrousos G., Corpas E., de Herder W.W., Dhatariya K., Dungan K., Hofland J. (2000). Lipid and Lipoprotein Metabolism in Liver Disease. Endotext.

[44] Cohen D.E., Fisher E.A. (2013). Lipoprotein metabolism, dyslipidemia, and nonalcohlic fatty liver disease. Semin. Liver Dis..

[45] Vargas M., Cardoso Toniasso S.C., Riedel P.G., Baldin C.P., Dos Reis F.L., Pereira R.M., Brum M.C.B., Joveleviths D., Alvares-da-Silva M.R. (2024). Metabolic disease and the liver: A review. World J. Hepatol..

[46] Lichtenstein A.H., Van Horn L. (1987). Very low-fat diets. Circulation.

[47] Skulas-Ray A.C., Wilson P.W., Harris W.S., Brinton E.A., Kris-Etherton P.M., Richter C.K., Jacobson T.A., Engler M.B., Miller M., Robinson J.G. (2019). Omega-3 fatty acids for the management of hypertriglyceridemia: A Science Advisory from the American Heart Association. Circulation.

[48] Neeli I., Siddiqi S.A., Siddiqi S., Mahan J., Lagakos W.S., Binas B., Gheyi T., Storch J., Mansbach C.M. (2007). Liver fatty acid-binding protein initiates budding of pre-chylomicron transport vesicles from intestinal endoplasmic reticulum. J. Biol. Chem..

