# Supplementary material for: Fenofibrate Treatment Inhibits Very-Low-Density Lipoprotein Transport Vesicle Formation by Reducing Sar1b Protein Expression
Source: Int J Mol Sci. 2025 May 15;26(10):4720. doi: 10.3390/ijms26104720 (PMC12111837; doi:10.3390/ijms26104720)
Supplement: Supplementary file 1 [file ijms-26-04720-s001.zip › ijms-3553846-supplementary.pdf]

Supplemental Figure S1

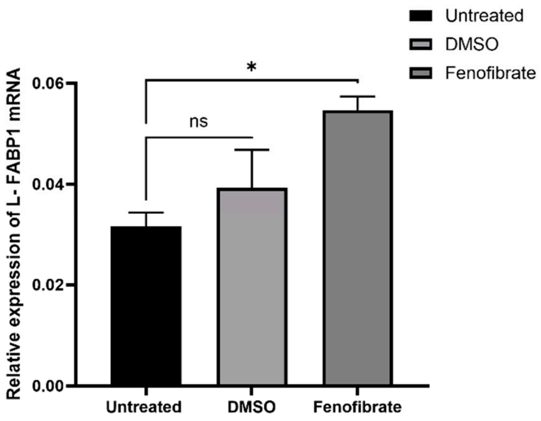

Supplemental Figure S1. Fenofibrate treatment enhances the L-FABP mRNA levels. RT-qPCR assay depicting relative levels of L-FABP mRNA in HepG2 cells under conditions of untreated, DMSO and Fenofibrate treated. The date are representative of mean ± SD of triplicates of two independent experiments. (* $p = 0.0391$)
